# Profiling of the tumor-associated microbiome in patients with hepatocellular carcinoma

**DOI:** 10.1186/s13099-025-00727-y

**Published:** 2025-07-10

**Authors:** Christian Schulz, Ramiro Vilchez-Vargas, Elif Öcal, Nadine Koch, Daniel Puhr-Westerheide, Lu Fornés Burnell, Heidrun Hirner-Eppeneder, Julia Benckert, Maciej Pech, Peter Reimer, Chris Verslype, Christiane Kuhl, Albert Tran, Jens Ricke, Peter Malfertheiner, Marianna Alunni-Fabbroni

**Affiliations:** 1 Department of Medicine II, LMU University Hospital, LMU Munich, Munich, Germany; 2 Department of Radiology, LMU University Hospital, LMU Munich, Marchioninistr. 15, 81377 Munich, Germany; 3 Department of Hepatology and Gastroenterology, Charité, Berlin, Germany; 4 Departments of Radiology and Nuclear Medicine, Otto-Von-Guericke University of Magdeburg, Magdeburg, Germany; 5 Department of Radiology, Karlsruhe Hospital, Karlsruhe, Germany; 6 Department of Hepatology and Digestive Oncology, University Hospital Gasthuisberg, Leuven, Belgium; 7 Department of Diagnostic and Interventional Radiology, University Hospital, RWTH Aachen University, Aachen, Germany; 8 Department of Immunology, Université De Nice Sophia-Antipolis, CHU De Nice, Nice, France; 9 Department of Gastroenterology, Hepatology and Infectious Diseases, Otto-Von-Guericke University of Magdeburg, Magdeburg, Germany

**Keywords:** Microbiota, Helicobacter pylori, Hepatocellular carcinoma, Liver, Interventional radiology

## Abstract

**Background:**

Tumor tissues have been shown to host a diverse array of bacteria, suggesting a link between the intratumoral microbiota and the development and progression of cancer. The aim of this explorative study was to perform microbiome analysis in liver tumor and to evaluate its relationship with cancer stage and survival outcome.

**Results:**

We conducted an exploratory study on a cohort of 20 hepatocellular cancer patients from the SORAMIC trial. Patients were divided into curative and palliative groups according to treatment type (local ablation, alone or combined with systemic therapy). The V1-V2 regions of *16 S rRNA* were sequenced starting from archival tissues. Amplicon Sequence Variants (ASVs) were taxonomically assigned to the upper (UGI) or lower (LGI) gastrointestinal tract. Bacteria were identified in both tumoral and non-tumoral tissues, showing higher diversity and correlation between diversity and shorter survival in the palliative group (*S. aureus p* < 0.05; *B. parvula p* < 0.01; *A. chinensis p* < 0.01). Both therapy groups were enriched with the genus *Bacilli*, including *Streptococcus spp.*, *Gemella haemolysans* and *Helicobacter pylori*, commonly found in UGI. The results suggested that among palliative patients and those with shorter survival, *G. haemolysans* was more prevalent, while *H. pylori* was more often found in curative patients with longer survival. However none of the results were significantly different (*p* > 0.05). A higher microbiome biodiversity was associated with an increased number of lesions (*Hoylesella*, *Agathobacter*, *Sphingobium*, *Cardiobacterium*, *Photobacterium* and *Serratia*, all with *p* < 0.01).

**Conclusions:**

The presence of bacteria, predominantly from communities of the UGI, suggests their translocation into liver tissue due to impaired barrier function of the upper gut or the ascending pathway along the biliary duct system. The intratumoral prevalence of bacteria with proinflammatory and oncogenic potential suggests their potential role in HCC pathomechanisms.

**Supplementary Information:**

The online version contains supplementary material available at 10.1186/s13099-025-00727-y.

## Background

Hepatocellular carcinoma (HCC) accounts for 75–85% of malignant liver tumors and is the third leading cause of cancer death. In the complex sequential cascade of hepatocarcinogenesis, which mostly evolves from chronic inflammation with cirrhotic transformation of the liver parenchyma, an important role has more recently been assigned to the gut microbiota and its detection in the tumor micromilieu [1–4]. The gut microbiota refers to the diverse community of microorganisms including bacteria and fungi, residing in the gastrointestinal tract [5]. This community of microorganisms supports health by modulating (among others) the digestion, the immune system function, and the defense against pathogens. It also produces compounds like short-chain fatty acids that strengthen the gut barrier and reduce inflammation. Diet, medications, and environment shape its composition, making it vital for overall health [6, 7]. Microbiota can nevertheless contribute also to tumorigenesis through several direct or indirect mechanisms. It has been shown that a microbial dysbiosis can drive cellular senescence [8], chronic inflammation, induce the production of carcinogenic metabolites [9], influence the tumor microenvironment [10] and contribute to drug resistance [11].

The potential routes of entry for the gut microbiota into the liver are the portal vein, through the draining of the intestine, and the bile duct system. Under normal conditions, bacteria modulate tissue regeneration and repair, contributing to maintaining liver homeostasis via a process known as the *gut‒liver axis* [12, 13]. However, microbiota dysbiosis can play a role in the inhibition of normal tissue regeneration and the induction of liver failure and cancer [14–17]. In the latter case, the presence of micriobiota in the tumor microenvironment (TME) can affect the mechanism of immunosurveillance [18], affecting the immune cell recruitment [19], suppressing the anti-tumor activity of the infiltrating cells and enabling the relase of pro-tumorigenic cytokines [10]. The presence of microbiota in the TME may facilitate disease progression, promote metastasis development and affect the response to therapy [20, 21]. Bacteria can drive tumor progression through various pathways, including DNA damage, the activation of oncogenic signals and the modulation of the immune response [22–27]. Recent research has also demontsrated the role of microbiome in cellular senescence [8]. One example is given by *Helicobacter pylori* which upregulates pathways such as NF-kB and p53-p21, accelerating cellular senescence. The release of a pro-inflammatory secretome by senescent cells has been shown to play a pro-tumorigenic effect of non-senescent cells. Also *Enterococcus* is recognized to be associated with tumorigenesis, especially through the activation of ROS production and the activation of protumorigenic pathways such as EGFR [4]. The characterization of the bacteria in patients with HCC could offer valuable insights into the role of the microbiota in this malignancy [21, 28–32]. The presence of bacteria colonizing the human gastrointestinal tract, such as *Helicobacter*, *Enterococcus*, *Bacteroides* or *Fusobacterium*, has already been shown in fresh HCC tissues, as well as in fresh adjacent tumor tissue [33]. However, the bacterial communities found in formalin fixed-paraffin embedded (FFPE) tissues differ considerably from those found in fresh samples, revealing that bacteria in FFPE samples are not frequently found in the human gastrointestinal tract [31, 34, 35]. Despite increasing evidence that the tumor microbiota may influence HCC progression and outcomes, significant knowledge gaps remain. Most studies have focused on gut or blood samples, while direct analysis of bacterial communities in HCC tumor tissue—especially using FFPE samples—has been limited by technical challenges and incomplete reference databases. Additionally, the heterogeneity of tumor-associated microbiota and unclear functional roles further complicate biomarker discovery. Therefore, systematic studies are needed to precisely characterize the tumor microbiota in relation to HCC stage and survival, ultimately aiding in the identification of clinically relevant microbial biomarkers. The aim of our explorative study was to characterize the bacterial microbiota in the tumor tissue of patients with HCC according to stage and overall survival (OS) as well as in nontumor FFPE tissue where available. We developed a systematic analysis pipeline incorporating detailed taxonomic annotation and a comprehensive database to enable precise identification of taxa in FFPE samples and aid in detecting potential biomarkers in HCC patients.

## Methods

### Ethical consideration

The present exploratory study is a substudy of the prospective, randomized controlled, multicenter phase II SORAMIC trial (EudraCT 2009-012576-27, NCT01126645), which was conducted in 12 countries in Europe and Turkey [36, 37]. The study was approved by the institutional review boards of all 38 participating centers and was conducted according to the ethical principles expressed in the Declaration of Helsinki. Written informed consent was obtained from all participants.

### Study population and tissue collection

The study population initially included 66 patients, 46 of whom were excluded because of no PCR product. Among the remaining 20 patients, 10 were diagnosed with HCC in the early and intermediate stages (curative group, defined by eligibility for local ablation in SORAMIC), and 10 were diagnosed with HCC at an intermediate or advanced stage (palliative group, defined by eligibility for locoregional combined with systemic treatment in SORAMIC). Within the palliative group, 1 patient presented with lung metastases, and 2 presented with lymph node metastases. In total, 23 FFPE blocks (16 tumor tissues and 7 nontumor tissues) were prepared from tumor tissues collected at baseline. From 3 patients in the curative group, tissues were taken from both the tumoral and adjacent areas. The classification of the tissues was performed by a board-certified pathologist.

### DNA extraction and amplicon library preparation

Genomic DNA was extracted from formalin-fixed, paraffin-embedded (FFPE) liver tissue samples via the QIAamp DNA FFPE Tissue Kit (Qiagen, Hilden, Germany) according to the manufacturer’s instructions. Briefly, 10 μm of each FFPE sample was cut via a standard microtome, after which the first 3 sections were discarded to avoid cross-contamination. After deparaffinization (3 min, 56 °C), proteinase K lysis (1 h, 56 °C, 1000 rpm) and incubation at 90 °C for 1 h to remove the cross-links, the samples were treated with RNase A (2 min, room temperature) and proteinase K (15 min, 65 °C). The DNA was subsequently bound to the columns, washed and eluted in 30 µl of elution buffer (Buffer ATE). The V1-V2 region of the bacterial *16 S rRNA* gene was amplified via PCR via Platinum™ SuperFi II PCR Master Mix (Thermo Fisher Scientific, Darmstadt, Germany) and the primers 27 F (5‘– AGRGTTHGATYMTGGCTCAG– 3‘) and 338R (5´TGCTGCCTCCCGTAGGAGT– 3‘) for 20 cycles as described previously [38]. The DNA concentration was measured via the Quant-iT™ PicoGreen™ dsDNA Assay (Thermo Fisher Scientific). The samples were pooled at equal molar concentrations before being purified (QIAquick PCR Purification, Qiagen) and sequenced on an Illumina MiSeq v3 with 300 bp paired-end sequencing. As negative controls, paraffin-only samples prepared at each recruitment center were used, as were all the reagents used for DNA extraction.

### Immunohistochemical analysis of tissue samples

From FFPE tumor blocks collected before therapy, 4 μm serial tissue sections were cut, dewaxed and rehydrated according to standard procedures (preheating at 60 °C; deparaffinization in Neo-Clear, Merck, Darmstadt, Germany; rehydration in a graded series of ethanol and distilled water) and stained with hematoxylin and eosin for determination of tumor and adjacent areas. For immunohistochemistry (IHC), the primary antibodies anti-LPS (mouse monoclonal, clone WN1 222-5, dilution 1:100, Hycult Biotech, Uden, The Netherlands) and anti-LTA (mouse monoclonal, clone 55, dilution 1:50, Hycult Biotech) were applied overnight at 4 °C, followed by incubation with the secondary antibody (goat anti-mouse IgG H&L/HRP, dilution 1:2000, Abcam, Cambridge, UK). A DAB substrate kit (DAB substrate kit, Cell Signaling Technology, Leiden, The Netherlands) was used as a chromogen. The sections were counterstained with Hemalaun (Merck), dehydrated, and mounted with Neo-Mount (Merck). The slides were scanned via an automated slide scanner (Pannoramic SCAN II, 3D HISTECH, Hungary) at 40x magnification. Identification of the cellular subtypes and bacteria was performed by a board-certified pathologist who was blinded to the patient outcome. The presence of defined LPS and LTA signals throughout the tumor tissue was evaluated as positive, whereas the complete absence of signals was evaluated as negative. Tissues with brown cytoplasmic staining in tumoral or immune cells were considered positive.

### Bioinformatic analysis

Paired fastQ files were analyzed with the dada2 package in R (v. 4.1.3) [39], and the count table for all 26 samples (including the negative controls with paraffin and reagents) was obtained. Sequences detected in the negative controls, independent of their relative abundances, were excluded from downstream analysis. After this first QC, one FFPE sample was discarded because no further reads were detected. From the remaining 23 FFPE samples, a total of 125,057 reads were obtained, with a median of 5,262 sequences, with a maximum of 17,830 sequences and a minimum of 10 sequences. The reads were grouped into 997 unique ASVs. ASVs were taxonomically annotated following two steps. At first an automatic annotation based on the silva database [40] was used. The second step was based on a manual annotation, using the NCBI database and comparing them exclusively against the type material database to define the discriminatory power of each sequence read [41]. Furthermore, on the basis of publicly available data [35, 42–44], all 997 ASVs were manually assigned into two groups according to their origins: paraffin origin (including environmental contamination and human skin contamination) and human origin (including the human upper gastrointestinal tract, the human lower gastrointestinal tract and potential human pathogens). Among the 997 ASVs, 526 were affiliated with human origin, and only these ASVs were considered for downstream analysis (Supplementary File - SF1). Additionally, a network of cooccurrences was constructed via Spearman’s rank correlation coefficient, with a cutoff of rho > 0.4 and a *p* value of < 0.05 after Benjamini‒Hochberg false discovery correction. Correlations were calculated via the psych and reshape2 packages in R. In summary, for the analysis of potential contaminations, we first considered the known habitat of each ASV, independently of its relative abundance. Each ASV detected in the cohort was annotated using BLAST against type strain databases, and only taxa previously reported in the human gastrointestinal tract were considered. Second, we applied a threshold of ≤ 5 for the node degree within the bacterial co-occurrence network and discussed only those genera meeting this criterion, assuming a scenario of low-level colonization and a correspondingly low probability of contamination. The network was visualized and analyzed via Cytoscape v. 3.10.1 [45, 46]. Due to the small cohort size, the only feasible statistical test was the indicator species analysis, which serves as a starting point for generating hypotheses regarding key genera or species potentially relevant in FFPE samples from HCC patients. Differences between predefined groups were assessed using an unsupervised hierarchical clustering using Bray-Curtis algorithm and indicator species analysis in PAST4 [47]. The extended Materials and Methods section is available in Supplementary File– SF9.

## Results

### Study population and characteristics

Within the study group, 50% of the patients (*n* = 10) were diagnosed with HCC in the early and intermediate stages and, in the frame of the SORAMIC study, were assigned to local ablation (curative group, BCLC stage A, *n* = 5; BCLC stage B, *n* = 5). The remaining 50% had HCC at an intermediate or advanced stage (assigned to palliative combined locoregional and systemic treatment: palliative group, BCLC stage B, *n* = 2; BCLC stage C, *n* = 8). The median age was 67.5 years (range 54–82), and 75% of the patients (*n* = 15) were male. Most of the patients had preserved liver function according to the Child‒Pugh class A (*n* = 19, 95%). Nine patients (45%) presented with diffuse disease (> 20 lesions); for the remaining 11 patients, the median number of lesions was 2 (range 1–4). The median size of the lesions was 50.8 mm (range 12–170 mm). Most of the patients had cirrhosis (*n* = 17, 85%) due to MASH (*n* = 7, 35%), chronic viral hepatitis (*n* = 6, 30%), alcohol abuse (*n* = 5, 25%) or cryptogenic hepatitis (*n* = 2, 10%). Within the curative group, 5 patients were treated with RFA + sorafenib, 4 with RFA + placebo, and 1 with sorafenib only. In the palliative group, 6 patients received selective internal radiation therapy (SIRT) + sorafenib, and 3 patients received sorafenib only. One patient in this group received best supportive care only. Tumor tissues were collected at baseline. The baseline characteristics of the patients are presented in Table 1.

**Table 1:** Baseline characteristics of the study population (*n* = 20)

Baseline Characteristics	*N* (% or range)	*N* (% or range)	*N* (% or range)
	All (*n* = 20)	Curative (*n* = 10)	Palliative (*n* = 10)
Age, mean (range)	67.5 (54–82)	65.0 (54–82)	70.1 (58–79)
Gender (male)	15 (75.0)	7 (70.0)	8 (80.0)
Cirrhosis (yes)	17 (85.0)	9 (90.0)	8 (80.0)
Etiology MASH Viral hepatitis Alcohol abuse Cryptogenic	7 (35.0) 6 (30.0) 5 (25.0) 2 (10.0)	3 (30.0) 2 (20.0) 4 (40.0) 1 (10.0)	4 (40.0) 4 (40.0) 1 (10.0) 1 (10.0)
Number of lesions (> 20 = DD) < 20 > 20	11 (55.0) 9 (45.0)	10 (100) 0 (0)	1 (10.0) 9 (90.0)
Max diameter largest lesion (mm) Median diameter lesions (mm)	170 50.8 (12–170)	90 28.4 (12–90)	170 80 (32–170)
Metastases (no)	17 (85.0)	10 (100.0)	7 (70.0)
Child‒Pugh A B	19 (95.0) 1 (5.0)	9 (90.0) 1 (10.0)	10 (100.0) 0 (0.0)
BCLC stage A B C	5 (25.0) 7 (35.0) 8 (40.0)	5 (50.0) 5 (50.0) 0 (0.0)	0 (0.0) 2 (20.0) 8 (80.0)
Therapy (Curative) RFA + Sorafenib RFA + Placebo Sorafenib only	5 (25.0) 4 (20.0) 4 (20.0)	5 (50.0) 4 (40.0) 1 (10.0)	0 (0) 0 (0) 3.0 (30.0)
Therapy (Palliative) SIRT + Sorafenib SIRT + Placebo Sorafenib only	6 (30.0) 0 (0.0) 4 (20.0)	0 (0.0) 0 (0.0) 1 (10.0)	6 (60.0) 0 (0.0) 3.0 (30.0)
Therapy (unknown)	1 (5.0)	0	0
Overall Survival ≥ 16 months < 16 months	8 (40.0) 12 (47.8)	6 (60.0) 4 (40.0)	2 (20.0) 8 (80.0)

Abbreviations: BCLC, Barcelona Clinic Liver Cancer; DD, diffuse disease; MASH, metabolic dysfunction-associated steatohepatitis; RFA, radiofrequency ablation; SIRT, selective internal radiation therapy

### Identification of gram-positive and gram-negative bacteria in tumoral, stromal and adjacent tissues

**Fig. 1:**
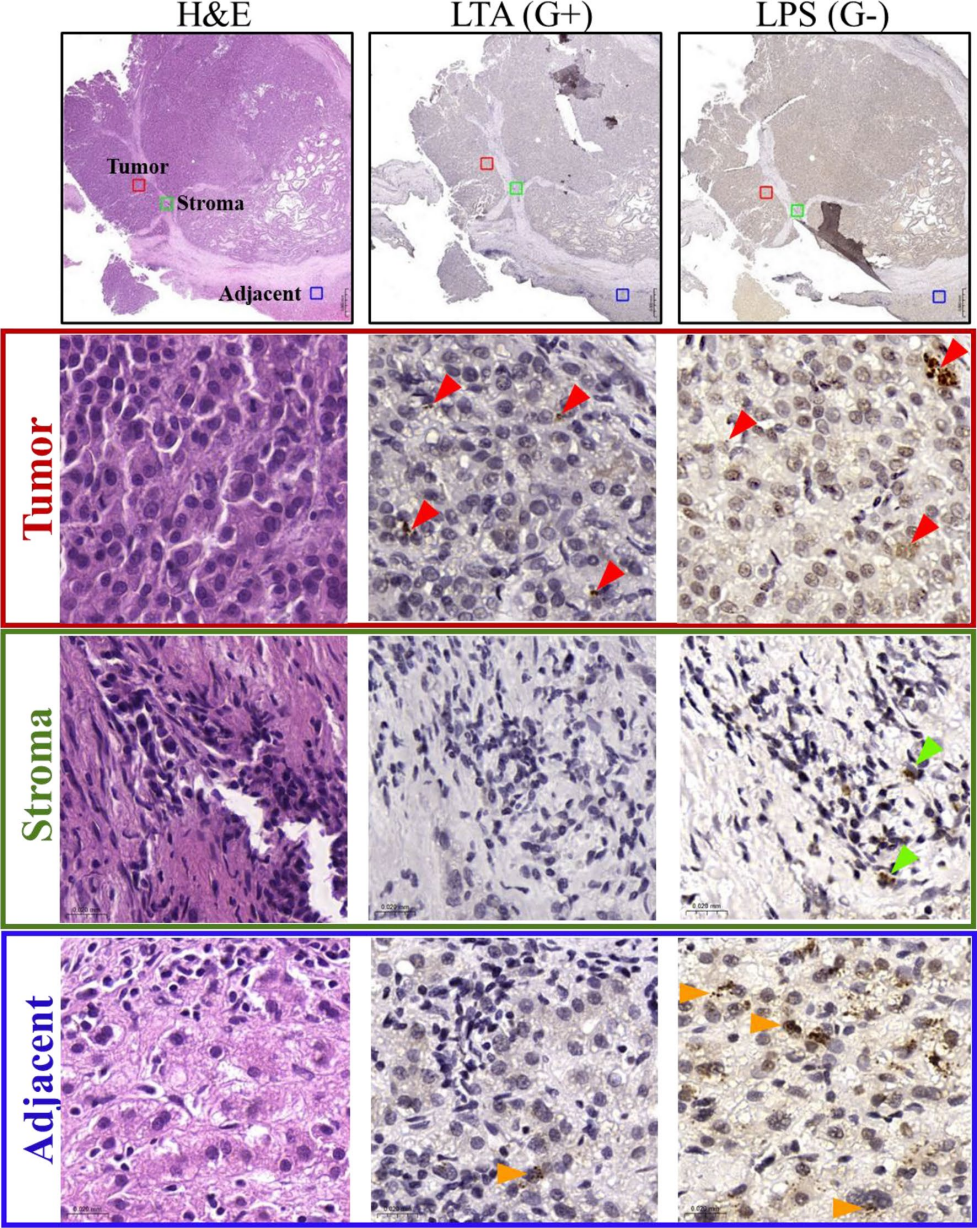
Representative images of the intratumor localization of bacteria in FFPE liver tissues. Images show the results of immunochemical staining for lipopolysaccharide (LPS) and lipoteichoic acid (LTA) in hepatocellular cancer tumor, stromal and adjacent tissues to detect gram-negative and gram-positive bacteria, respectively. Bacteria identified in tumor cells are denoted with red arrows, stromal cells with green arrows and hepatocytes with orange arrows. Scale bars, 0.060 mm and 0.020 mm. Magnification: 1x in the upper panels and 20x in the tumor, stroma and adjacent panels

The presence and distribution of bacteria were first evaluated in tumoral, stromal and adjacent tissues. Lipotheichoic acid (LTA) and lipopolysaccharide (LPS) are the markers used to detect gram-positive and gram-negative bacteria, respectively [48]. Pathological examination of the tissues confirmed the presence of gram-positive and gram-negative bacteria in tumor cells and in hepatocytes from adjacent, nontumoral liver tissues. In contrast, in stroma cells, only gram-negative bacteria were identified (Fig. 1).

### Pipeline for the analysis of the bacterial community in FFPE samples from the liver

**Fig. 2:**
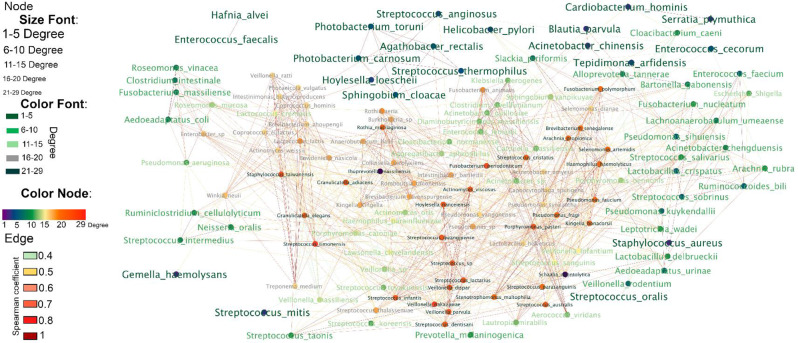
Co-occurrence bacterial network in FFPE liver tissues. Size fonts, color fonts and color nodes denote the level of degree (number of neighbors) of each node, and edge colors denote rho values. Level of significance: *p* value < 0.05 (correlations were calculated with Spearman’s rank correlation coefficient (rho ≥ 0.4) after false discovery rate correction via the Benjamin‒Hochberg procedure)

Despite removing all the sequences detected in the negative controls (including all reagents and paraffin without biological material), many ASVs, numerous with high relative abundance, were annotated to taxa that have never been described in the human gastrointestinal tract. Thus, the first step was to construct a database focused on the axis of the human liver and human gut, including all the ASVs detected in the cohort, and to assign the most likely environment to each of the 997 ASVs on the basis of publicly available NCBI data (SF1 and SF2). Whether an ASV was not previously described as inhabiting the human gut [30], it was considered an external contaminant, because of nonsterile paraffin or nonsterile environment during sample preparation [21, 28, 31]. Some ASVs could not be uniquely assigned to a single environment. For example, *Finegoldia magna*, which is typically a skin colonizer, has also been reported in the urinary tract microbiota [49]. In these cases, cobacterial communities within the same tissue were examined. For example, in sample SOR42, *Finegoldia magna* was detected alongside *Corynebacterium tuberculostearicum*, and both were classified as paraffin-associated contaminants. In the database, each sequence was analyzed individually and within the same FFPE sample, and its origin was determined to increase the degree of discrimination between the sources of the taxa. A total of 491 ASVs were confirmed to originate from the human gastro-intestinal (GI) tract (SF1), comprising 134 unique species and 66 genera (SF3). Among the five species with the highest frequency across the entire cohort, *Streptococcus mitis* was found in 52.1% of the patients; *Streptococcus oralis* and *Gemella haemolysans* were detected in 47.8% of the patients; *Haemophilus parainfluenzae* was detected in 43.4% of the patients; *Enterococcus cecorum*, *Streptococcus toyakuensis* and *Veillonella massiliensis* were detected in 30.4% of the patients; and *Lawsonella clevelandensis*, *Staphylococcus aureus* and *Streptococcus koreensis* were detected in 26.0% of the patients (SF3). Notably, most of the detected bacteria belong to the human upper gastrointestinal (UGI) tract, with the exception of *Enterococcus cecorum*. Next, we investigated the interactions (or cooccurrences) among the detected bacteria to determine whether the colonization could be attributed to a single bacterium (no interactions), a small number of bacteria (sparse interactions), or polymicrobial colonization involving a community (multiple interactions). The abundances of all the colonizers were correlated within each individual sample, and a network of co-occurrences was generated. Hence, a network with 134 bacterial species, comprising 921 interactions, was built (Fig. 2, SF4), with only positive correlations detected (no negative interactions were found). The degree (number of neighbors) of each taxon detected in the cohort was used as a marker to indicate how many interactions a single bacterium had within the sample. Members of the network were grouped according to their degree level, which varied from 1 (*Streptococcus oralis*,* Enterococcus faecalis*,* Hafnia alvei and Gemella haemolysans* with 1 interaction) to 29 neighbors (*Ihuprevotella massiliensis* and *Schaalia odontolytica* with 29 interactions).

### Microbiome composition of FFPE blocks from cancerous and adjacent tissues

**Fig. 3:**
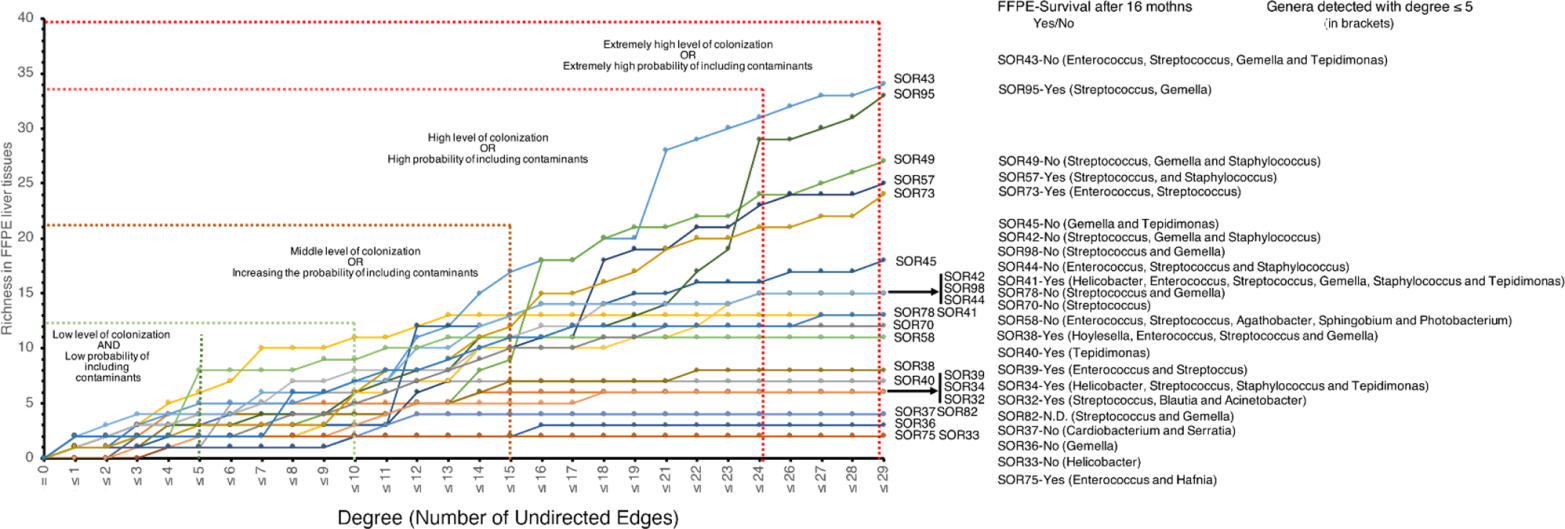
Richness as a function of the degree (number of neighbors) of the different members of the bacterial community network. Genera detected with a degree of ≤ 5 in each FFPE liver tissue sample are indicated in brackets

**Fig. 4:**
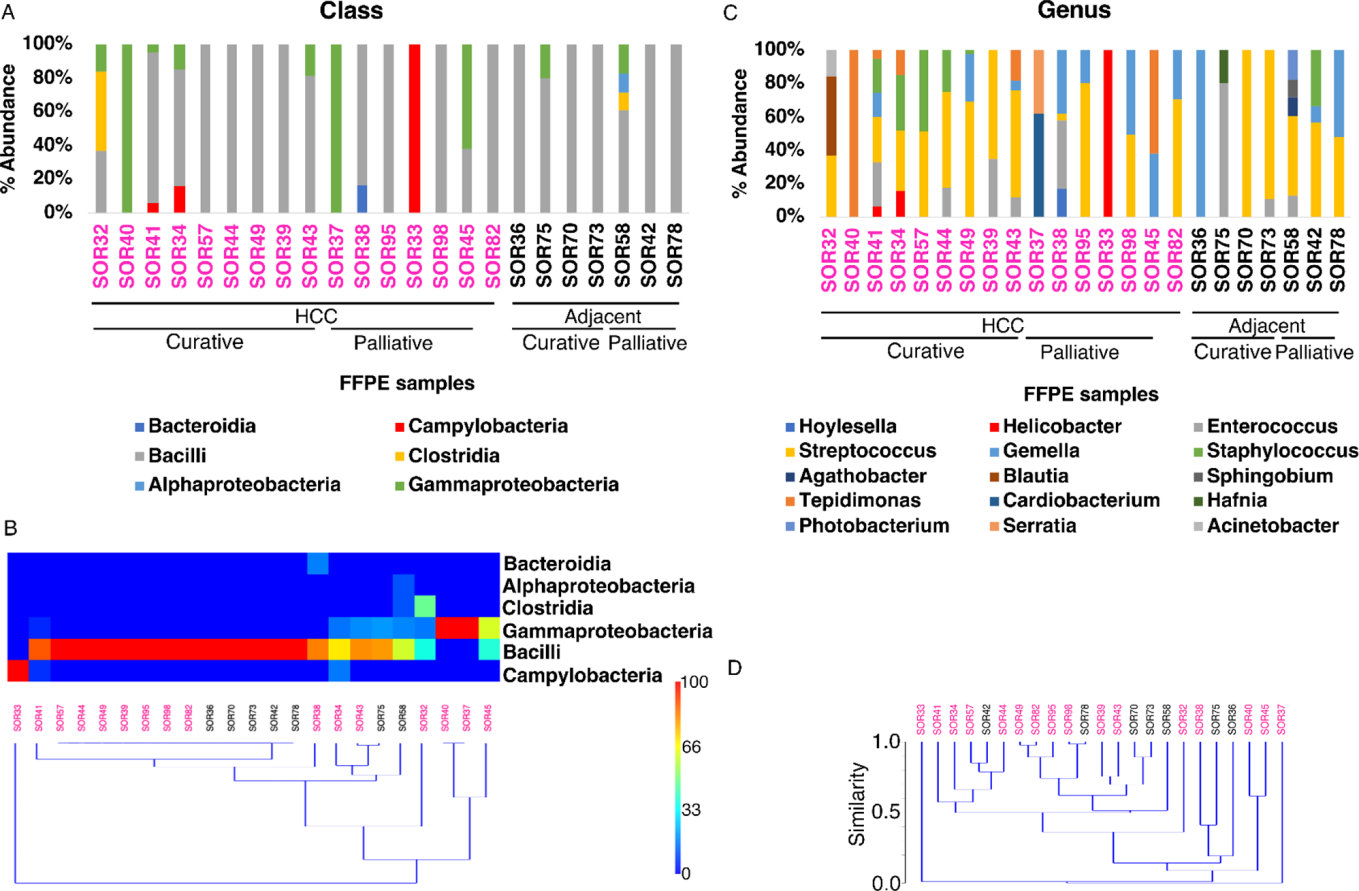
Overall bacterial diversity (top) and unsupervised hierarchical clustering of prevalent taxa detected in tumor (HCC) and adjacent tissues via the Bray‒Curtis dissimilarity index (bottom). Both the top and bottom panels display the results from FFPE liver tissues at the taxonomic ranks of class (**A**, **B**) and genus (**C**, **D**). Bootstrapping analysis is reported in SF7

**Fig. 5:**
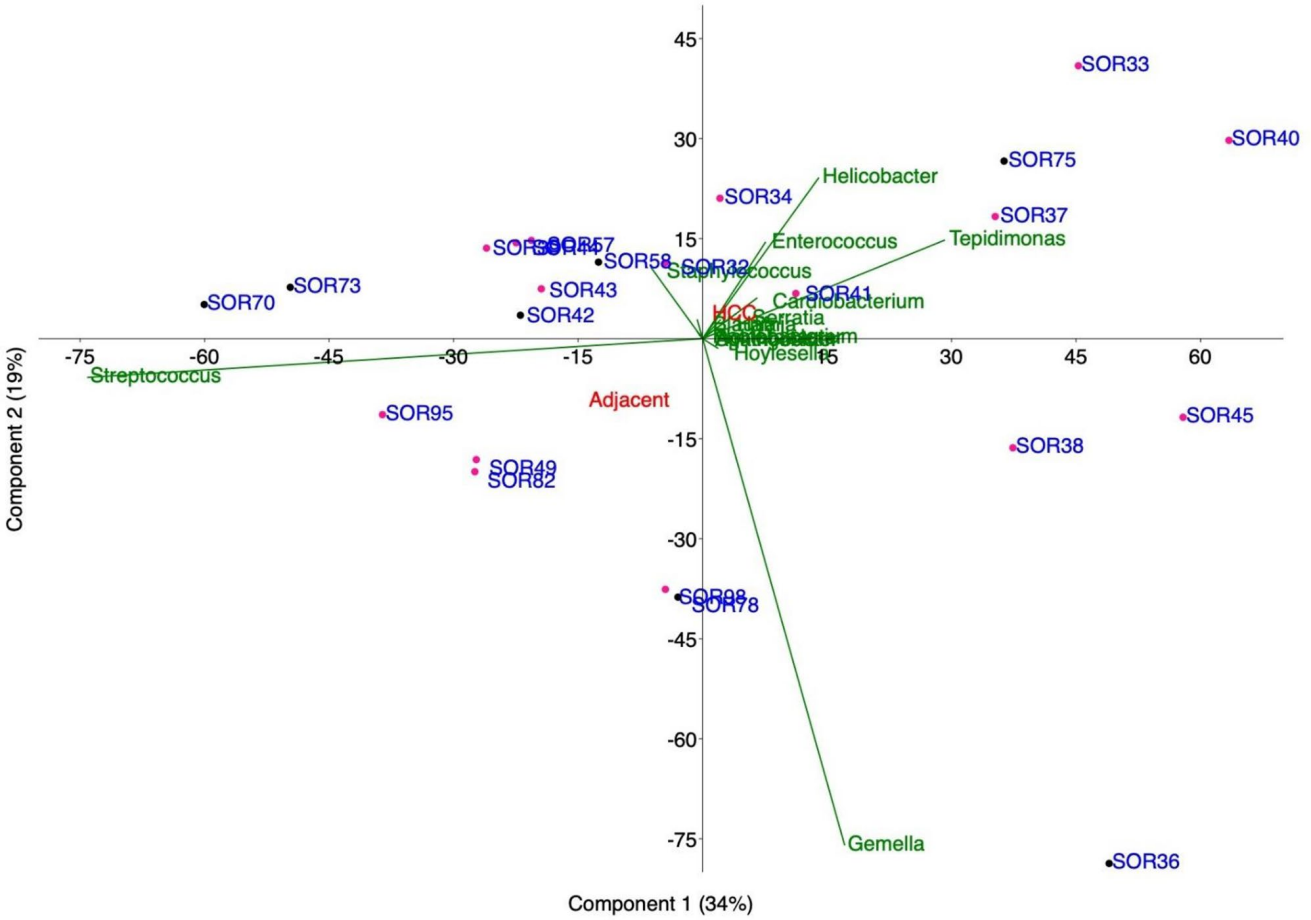
Principal component analysis (PCA). The analysis was performed starting from tumor (red) and adjacent (black) FFPE liver tissues, with each group name positioned at its respective data centroid

The richness, defined as a function of the degree of each member within the bacterial community, was analyzed (SF5). We considered the likely scenario that a lower degree of interactions indicated a lower level of bacterial colonization in FFPE liver tissues. In this way the probability of including contaminants in the dataset was decreased. Conversely, an increase in the degree of interactions could have been associated with a significant increase in bacterial colonization levels. However, this may also increase the likelihood of introducing exogenous contaminants into the dataset (Fig. 3). For the following analysis, low cobacterial presence (degree ≤ 5) was considered, reflecting the scenario of low bacterial colonization in FFPE liver tissues. Through this in silico analysis, 20 bacterial species across the entire cohort were identified. At the taxonomy rank of class (SF6), the prevalence of Bacilli in tumors as well as in adjacent tissues was clear, with 52% of the samples entirely colonized by this class. Gammaproteobacteria were identified in 35% of the samples and were uniquely detected in 2 samples (samples SOR40 and SOR37). At the genus level, low similarity among tissues was observed (Fig. 4). FFPEs had a median species richness of 3, with a minimum of 1 (samples SOR40, SOR33 and SOR36) and a maximum of 8 (sample SOR58) (Fig. 3). When the taxa at the genus level were analyzed, the median richness decreased to 2, primarily because *Streptococcus* (comprising species such as *S. anginosus*, *S. mitis*, *S. oralis*, and *S. thermophilus*) was the genus with the highest prevalence in the entire cohort (Fig. 4 and SF3). Notably, *Streptococcus* was present in 17 out of 23 FFPE samples. Interestingly, in four samples, only a single genus was detected: *Tepidimonas* (affiliated exclusively with *T. arfidensis*) in SOR40; *Helicobacter* (affiliated exclusively with *H. pylori*) in SOR33; *Gemella* (affiliated exclusively with *G. haemolysans*) in SOR36; and *Streptococcus* (affiliated with *S. mitis* and *S. oralis*) in SOR70. With the exception of *T. arfidensis*, whose origin remains unknown, all are well-known colonizers of the human UGI tract [43]. In conclusion, bacteria from the UGI tract were detected to a greater extent; however, *Enterococcus* (affiliated with *E. faecalis* and *E. cecorum*), *Agathobacter rectalis* (formerly named *Eubacterium rectale* [50]) and *Blautia parvula*, which are usually more abundant in the lower GI (LGI) tract, were also detected but to a lesser extent. When the bacterial communities of HCC and adjacent tissues were compared, principal component analysis (PCA) revealed that HCC tissues tended to cluster with higher abundances of certain genera (for example, *Helicobacter*, *Tepidomonas*, and *Enterococcus*), whereas adjacent tissues were more associated with *Streptococcus*, *Staphylococcus*, and *Gemella* (Fig. 5). However, none of these genera were exclusively found in either tissue type.

### Associations between the liver microbiome and the clinical characteristics of patients

**Fig. 6:**
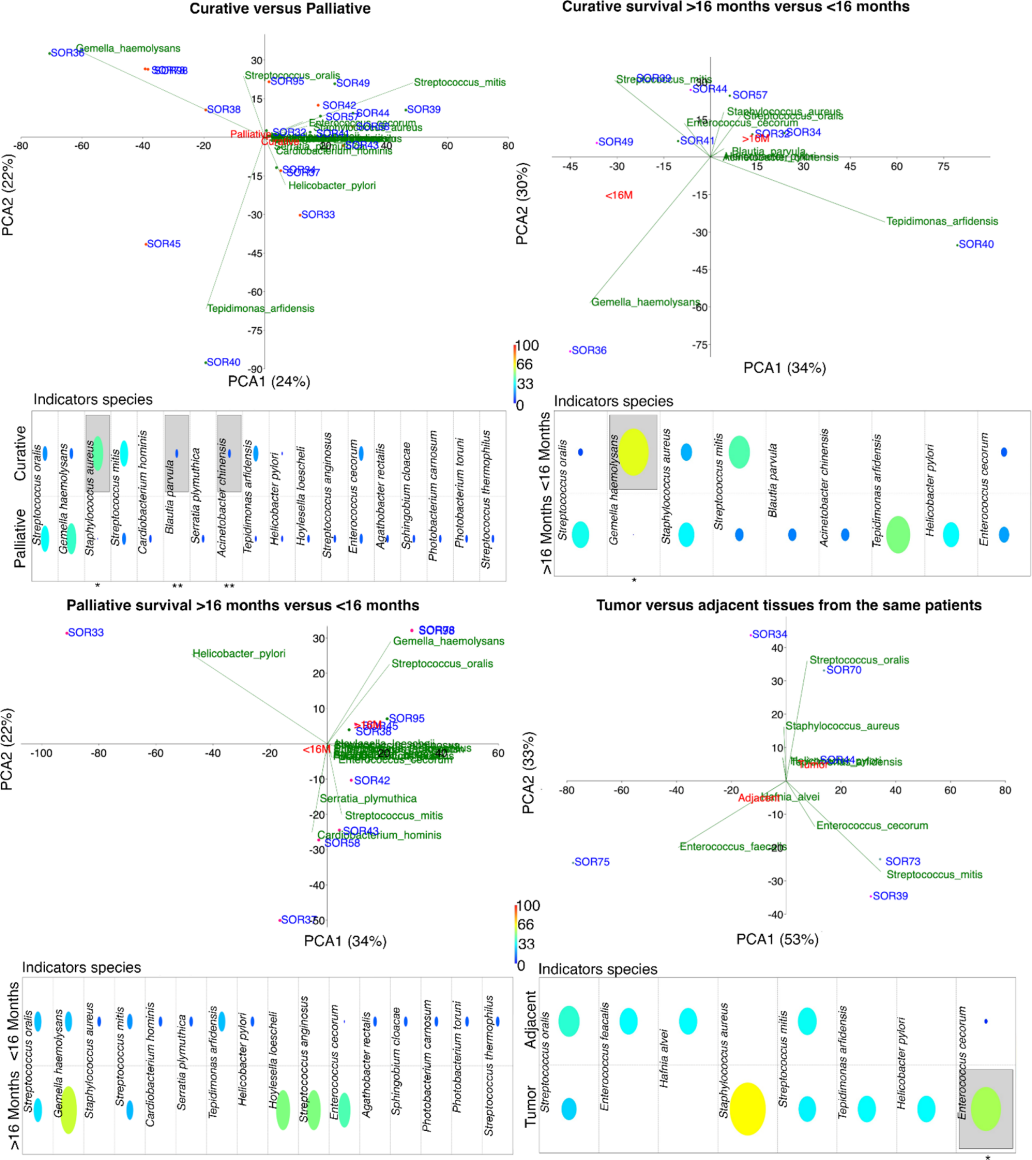
Principal component analysis (PCA) and indicator species analysis (ISA). The analysis was performed on FFPE liver tissues considering comparisons of the following clinical characteristics: curative vs. palliative (top left), survival > 16 months vs. < 16 months in the curative group (top right) and in the palliative group (bottom left), respectively, and tumor vs. adjacent tissues (bottom right). Level of significance: significant differences in the abundances of bacterial species within groups are indicated by gray boxes and marked with * for *p* < 0.05 and ** for *p* < 0.001 (differences between predefined groups were assessed using an unsupervised hierarchical clustering using Bray-Curtis algorithm and indicator species analysis)

To reveal the associations between the liver microbiome and the clinical characteristics of patients, we performed statistical analyses including the following clinical parameters: HCC stage (curative vs. palliative), survival within the curative group (≥ 16 months or < 16 months), survival within the palliative group (≥ 16 months or < 16 months) and malignancy (tumor tissue vs. adjacent tissue) (Fig. 6). Additional parameters were OS, BCLC stage (A, B, or C), number of lesions (group A if < 5 lesions vs. group B if ≥ 5 lesions) and level of alpha-fetoprotein (group A if < 200 ng/mL vs. group B if ≥ 200 ng/mL) (SF7). Neither the richness nor the Shannon, Simpson, and Pielou indices nor the PERMANOVA and Mann‒Whitney tests revealed any significant differences in any of the considered parameters (see Supplementary SF10). However, PCA and indicator species analysis identified specific species potentially associated with certain groups. For example, a greater number of species were detected in the palliative group than in the curative group, as well as in the palliative group, with shorter survival than in those with longer survival. The results did not allow us to assign any specific bacteria to a particular group conclusively. However, *Gemella haemolysans* was more abundant in the curative group with short survival and in both palliative groups, whereas contradictory findings were observed with *Helicobacter pylori*, which was more abundant in the curative group with long survival but was present only in the palliative group with short survival.

Overall, *Helicobacter pylori* and *Enterococcus cecorum* were more abundant in all groups with survival ≥ 16 months, whereas *Hoylesella loescheli* and *Streptococcus anginosus* were more abundant in the palliative care group, with ≥ 16 months of survival (Fig. 6 and SF7). Notably, greater biodiversity was detected in samples collected from patients with more than 5 lesions than in the other samples. These included *Hoylesella*, *Agathobacter*, *Sphingobium*, *Cardiobacterium*, *Photobacterium* and *Serratia*, all with *p* < 0.01 (SF7).

### Comparative analysis of bacterial composition in tumor and adjacent tissue samples

Finally, a comparative analysis of the bacterial composition of tumors and adjacent FFPEs from the same patient was performed. SOR44 (tumor) and SOR70 (adjacent) samples were collected from a 68-year-old woman with stage B HCC, a curative group, and MASH etiology who died within 16 months after sample collection (patient 62). SOR39 (tumor) and SOR73 (adjacent) samples were instead collected from a 66-year-old woman with stage A HCC, a curative group with viral etiology, who survived beyond 16 months after sample collection (patient 15). Patient 62 presented high immune infiltration in both tissues (tumor and adjacent tissue), whereas in the tissues collected from patient 62, 15 immune cells were poorly represented (SF8). Considering polybacterial colonization in the FFPE tissue samples, the richness of SOR44, SOR70, SOR39, and SOR73 was 15, 12, 6, and 24, respectively, with 135 bacterial interactions. However, when the analysis was restricted to a degree ≤ 5, the richness drastically decreased to 4, 2, 2, and 3 (SF5). Assuming low colonization in these samples, the bacterial landscape detected belonged exclusively to the class Bacilli, which included four genera: *S. oralis* and *S. mitis*, *S. aureus* and *E. cecorum*.

## Discussion

In this explorative study of tumor tissues obtained from patients with early- and advanced-stage HCC, we identified a heterogeneous microbiota profile, where the predominant bacteria in both tumor and adjacent tissues were those typically found in the UGI tract [43]. Although the small cohort size did not permit the use of more robust statistical methods, the application of PCA and indicator species analysis allowed the identification of several genera and species that may be associated with the variables under investigation. The results were consistent with those of prior studies [21] and highlight marked dysfunction in the gut‒liver axis, especially the UGI‒liver connection. Bacilli was the most relevant genus identified. In this class and across all species, the most notable and unexpected findings were four species belonging to the genus *Streptococcus*: *S. mitis*, *S. oralis*, *S. thermophilus* and *S. anginosus*. All of them are considered viridans group streptococci (VGS), with *S. mitis* and *S. oralis* being the predominant species belonging to *Streptococcus*, which causes severe clinical disease in cancer patients [51]. Furthermore, a recent report indicated that the secretion of antigens by *S. anginosus* is a proinflammatory mechanism contributing to the pathogenesis of several cancers, including HCC [52]. It should be noted that while immunohistological examination of the tissues revealed the presence of both Gram-positive and Gram-negative bacteria, our dataset indicates that the majority of Gram-negative bacteria were ultimately classified as contaminants (e.g., *Acinetobacter lwoffii*, which is commonly found on the skin). It is important to acknowledge the potential limitations of immunohistochemical identification of bacterial components in FFPE tissues. In particular, the use of anti-LPS antibodies to identify Gram-negative bacteria may yield false-positive results due to the persistence of LPS molecules in tissue. LPS is a structurally stable component of the outer membrane of Gram-negative bacteria and can remain in tissues long after the bacteria themselves have died and lysed. Consequently, positive LPS staining does not necessarily indicate the presence of intact or viable bacteria but may instead reflect residual bacterial debris. This biological persistence may account for the observed discrepancy between broad IHC detection of Gram-negative bacteria and their classification as likely contaminants in sequencing data. Furthermore, non-specific binding of antibodies, especially in inflamed or necrotic tissue regions, may contribute to background staining. To address these challenges and more accurately interpret bacterial presence in FFPE samples, multimodal validation using complementary techniques such as fluorescence in situ hybridization (FISH) or 16 S rRNA-targeted probes would be advisable to confirm the identity and spatial distribution of bacteria and reduce the risk of overinterpretation based on IHC alone.

Direct comparisons between tumor and adjacent nontumor tissues revealed the presence of *H. pylori* exclusively within the tumoral compartment. Although the liver is as atypical niche for *H. pylori* [53], the detection of DNA isolated from this species in both palliative and curative HCC patient groups aligns with previous findings [54, 55]. Additionally, the comparison between curative and palliative patients’ group identified a larger abudance of S. *aureus* in the first group. Moreover, the *S. aureus* abundance was greater in patients whose OS exceeded 16 months. *S. aureus* can be linked to chronic inflammation, which can foster a pro-tumorigenic microenvironment, as well as to acute inflammation which on the contrary promotes an anti-tumorigenic microenvironment [56]. Our results in HCC need therefore further clarification. In few patients we were able to analyse the microbiome in tumoral and adjacent tissues. Notably, bacteria were detected in both compartments, suggesting damage to the entire organ [51, 57–72]. The comparison between tumor and nontumor tissues also highlights tissue tropism [73]. For example, *Campylobacteria* and *Bacteroida* were exclusively identified within tumor tissues, whereas *Alphaproteobacteria* were detected only in nontumor regions. Additionally, some tissues were colonized by a single species, whereas others contained multiple species. Given the small cohort size, we must consider this result as preliminary. However, tissue tropism likely plays a role in liver pathogenesis, and a larger cohort study could clarify its potential correlation with clinical outcome. The data used in this study were obtained from archival tissues. Sequencing and taxonomic annotation revealed numerous sequences unlikely to represent genuine liver colonizers, as these species are absent in human UGI/LGI tracts. To further analyze this discrepancy, we developed a novel methodology, yielding two key findings: in certain FFPEs (e.g., SOR75), species richness remained stable, implying limited infiltration; in others (e.g., SOR43, SOR95), species richness increased significantly, suggesting either multi-bacterial invasion or possible contamination. Currently, there are insufficient available data to distinguish between these two results. Although FFPE samples are among the largest and most valuable resources in biobanking, it is important to acknowledge that, in most cases, they were collected and preserved under non-sterile conditions. As a result, microbiome analysis of these samples does not follow a straightforward pipeline, and careful consideration must be given to the potential origin of the detected bacterial species. Furthermore, due to the preservation and DNA extraction protocols, some underestimation of high GC content DNA sequences cannot be entirely excluded [74]. Taking the previous considerations into account, it is still noteworthy that both SOR43 and SOR95, each with a richness of approximately 35, were associated with necrotic liver tissue (data not shown). Further research is needed to explore more comprehensively the possibility of an association between bacterial richness and necrosis. Moreover, new models are required to elucidate whether bacteria are the cause or the consequence of hepatocellular carcinoma linking the gut-liver axis, similar to the zebrafish model [75], which has recently been proposed as a model for investigating the gut–brain axis [75, 76]. Recent research has highlighted the significant impact of microbial communities on liver health and disease progression. The influence of certain bacteria on the liver microenvironment is increasingly well understood. For instance, *S. mitis* [77] has been implicated in the development of pyogenic liver abscesses, which can compromise local immune defenses and create a milieu more susceptible to malignant transformation. By weakening immune surveillance, these abscesses may facilitate the emergence and progression of cancerous lesions. Additionally, *H. pylori* has been shown to induce significant liver injury by triggering robust inflammatory responses [55]. In the context of chronic liver diseases such as cirrhosis, this persistent inflammation can accelerate cancer progression and worsen patient outcomes. Collectively, these findings underscore the pivotal role of microbial factors in shaping the liver microenvironment and influencing the trajectory of liver disease and hepatocarcinogenesis. On another front, although dysbiosis has been strongly associated with carcinogenesis, microbiome-based therapies have demonstrated considerable potential in cancer treatment [5]. For example, fecal microbiota transplantation and the administration of live probiotics are well-established strategies in oncology [78]. In HCC, however, few studies have focused specifically on probiotic interventions. A retrospective study involving 1,267 patients with hepatitis B virus (HBV) infection undergoing antiviral therapy showed that probiotic use was associated with a reduced risk of developing HCC [79]. Furthermore, preclinical studies in animal models have demonstrated that probiotics can inhibit tumor growth and reduce tumor size by modulating the immune response [80]. Overall, probiotics exert beneficial effects by modulating gut-liver axis signaling, suppressing pro-tumorigenic inflammation, and reducing HCC risk in a dose-dependent manner among high-risk populations. While further clinical validation is required, probiotics represent a safe, cost-effective adjunct to standard HCC therapies. We acknowledge some limitations in the current study, which also highlight important opportunities for future improvement. The size of the cohort was small; therefore, validation studies are necessary to confirm these preliminary results. The nature of FFPE samples presents inherent challenges, as they typically yield low DNA content. In our case, successful PCR amplification was achieved in approximately 30% of the total samples. Then, the FFPE samples are not usually collected or preserved under sterile conditions, which may influence microbial profiles. Nonetheless, the ability to extract and analyze microbial DNA from such archived material demonstrates the feasibility of microbiome studies in FFPE tissues and opens the door for future methodological optimization and broader applications, even in retrospective clinical cohorts.

## Conclusions

Understanding the influence of the “dysbiotic” microbiome in liver carcinogenesis is crucial and presents an opportunity to leverage microbiome profiling as a predictive tool for evaluating response to oncological immunotherapy. Furthermore it may direct research towards the modulation of microbiota in the therapy of malignant liver disease.

## Electronic supplementary material

Below is the link to the electronic supplementary material.

Supplementary Material 1

Supplementary Material 2

Supplementary Material 3

Supplementary Material 4

Supplementary Material 5

Supplementary Material 6

Supplementary Material 7

Supplementary Material 8

Supplementary Material 9

Supplementary Material 10

## Data Availability

The datasets used and/or analyzed during the current study are available from the corresponding author on reasonable request.

## Abbreviations

ASV: Amplicon Sequence Variant
BCLC: Barcelona clinic liver cáncer
FFPE: Formalin fixed-paraffin embedded
HCC: Hepatocellular carcinoma
IHC: Immunohistochemistry
LGI: Lower gastro-intestinal tract
LPS: Lipopolysaccharide
LTA: Lipotheichoic acid
MASH: Metabolic dysfunction-associated steatohepatitis
OS: Overall survival
PCA: Principal component analysis
RFA: Radiofrequency ablation
SIRT: Selective internal radiation therapy
TME: Tumor microenvironment
UGI: Upper gastro-intestinal tract
