# Supplementary material for: Profiling of the tumor-associated microbiome in patients with hepatocellular carcinoma
Source: Gut Pathog. 2025 Jul 10;17:53. doi: 10.1186/s13099-025-00727-y (PMC12243435; doi:10.1186/s13099-025-00727-y)
Supplement: Supplementary file 2 — Supplementary Material 2 [file 13099_2025_727_MOESM2_ESM.pdf]

Taxon	Ref	Habitat	Contamination described in
Agrobacterium (e.g. *fabrum*, *rosae*)	[1]	Soil	this study
Rhodococcus (*qingshengii*)	[2]	Soil/Plants	this study
Anoxybacillus (e.g. *eryuanensis*)	[3]	Geothermal spring	this study
Bacillus (e.g *coagulans*, *suaedae*, *thuringiensis*)	[4]	Evaporated milk/soil	this study
Deinococcus (e.g. *geothermalis*)	[5]	Geothermal spring	this study
Geobacillus (*stearothermophilus*)	[6]	Geothermal spring/Soil	this study
Moraxella (*tetraodonis*)	[7]	Fresh water	this study
Paracoccus (e.g. *carotinifaciens*, *marinus*, *panacisoli*)	[8-10]	Soil/sea water	this study
Rhizobium (e.g. *oryzihabitans*)	[11]	Soil/rice roots	[12]
Pseudoxanthomonas (*taiwanensis*)	[13]	Geothermal spring	this study
Corynebacterium tuberculostearicum	[14, 15]	Human skin	this study
Cutibacterium acnes formerly *Propionibacterium acnes*	[16]	Human skin	this study
Finegoldia magna	[17]	Human skin	this study
Brucella lupini	[1]	*Lupinus* roots	this study
Staphylococcus epidermidis	[18]	Human skin	this study
Acinetobacter (e.g. *lwoffii*, *junii*)	[19]	Human skin/soil	[12, 20]

Supplementary File 2. Representative taxa manually assigned as exogenous to FFPE liver tissue from HCC patients

Supplementary Bibliography

1. Msaddak, A., et al., Lupin, a Unique Legume That Is Nodulated by Multiple Microsymbionts: The Role of Horizontal Gene Transfer. Int J Mol Sci, 2023. 24(7).
2. Kuhl, T., et al., Genome-Based Characterization of Plant-Associated Rhodococcus qingshengii RL1 Reveals Stress Tolerance and Plant-Microbe Interaction Traits. Front Microbiol, 2021. 12: p. 708605.
3. Zhang, C.M., et al., Anoxybacillus tengchongensis sp. nov. and Anoxybacillus eryuanensis sp. nov., facultatively anaerobic, alkalitolerant bacteria from hot springs. Int J Syst Evol Microbiol, 2011. 61(Pt 1): p. 118-122.
4. Aldaby, E.S.E., et al., Microalgal upgrading of the fermentative biohydrogen produced from Bacillus coagulans via non-pretreated plant biomass. Microb Cell Fact, 2023. 22(1): p. 190.

5. Ferreira, A.C., et al., Deinococcus geothermalis sp. nov. and Deinococcus murrayi sp. nov., two extremely radiation-resistant and slightly thermophilic species from hot springs. Int J Syst Bacteriol, 1997. 47(4): p. 939-47.
6. Burgess, S.A., et al., Insights into the Geobacillus stearothermophilus species based on phylogenomic principles. BMC Microbiol, 2017. 17(1): p. 140.
7. Das, L. and S.K. Das, Moraxella tetraodonis sp. nov., isolated from freshwater pufferfish (Tetraodon cutcutia) skin. Arch Microbiol, 2022. 204(7): p. 389.
8. Nguyen, N.L., et al., Paracoccus panacisoli sp. nov., isolated from a forest soil cultivated with Vietnamese ginseng. Int J Syst Evol Microbiol, 2015. 65(Pt 5): p. 1491-1497.
9. Rai, A., et al., Paracoccus aeridis sp. nov., an indole-producing bacterium isolated from the rhizosphere of an orchid, Aerides maculosa. Int J Syst Evol Microbiol, 2020. 70(3): p. 1720-1728.
10. Lin, P., et al., Paracoccus hibiscisoli sp. nov., isolated from the rhizosphere of Mugunghwa (Hibiscus syriacus). Int J Syst Evol Microbiol, 2017. 67(7): p. 2452-2458.
11. Hardoim, P.R., et al., Dynamics of seed-borne rice endophytes on early plant growth stages. PLoS One, 2012. 7(2): p. e30438.
12. Borgognone, A., et al., Performance of 16S Metagenomic Profiling in Formalin-Fixed Paraffin-Embedded versus Fresh-Frozen Colorectal Cancer Tissues. Cancers (Basel), 2021. 13(21).
13. Chen, M.Y., et al., Pseudoxanthomonas taiwanensis sp. nov., a novel thermophilic, N2O-producing species isolated from hot springs. Int J Syst Evol Microbiol, 2002. 52(Pt 6): p. 2155-61.
14. Ahmed, N.M., et al., Genomic characterization of the C. tuberculostearicum species complex, a ubiquitous member of the human skin microbiome. bioRxiv, 2023.
15. Altonsy, M.O., et al., Corynebacterium tuberculostearicum, a human skin colonizer, induces the canonical nuclear factor-kappaB inflammatory signaling pathway in human skin cells. Immun Inflamm Dis, 2020. 8(1): p. 62-79.
16. Dreno, B., et al., Acne microbiome: From phyla to phylotypes. J Eur Acad Dermatol Venereol, 2024. 38(4): p. 657-664.
17. McCarthy, S., et al., Altered Skin and Gut Microbiome in Hidradenitis Suppurativa. J Invest Dermatol, 2022. 142(2): p. 459-468 e15.
18. Yoon, S., et al., Detection of anaerobic and aerobic bacteria from commercial tattoo and permanent makeup inks. Appl Environ Microbiol, 2024. 90(7): p. e0027624.

19. Fyhrquist, N., et al., Acinetobacter species in the skin microbiota protect against allergic sensitization and inflammation. J Allergy Clin Immunol, 2014. 134(6): p. 1301-1309 e11.
20. Eisenhofer, R., et al., Contamination in Low Microbial Biomass Microbiome Studies: Issues and Recommendations. Trends Microbiol, 2019. 27(2): p. 105-117.
