# Supplementary material for: Profiling of the tumor-associated microbiome in patients with hepatocellular carcinoma
Source: Gut Pathog. 2025 Jul 10;17:53. doi: 10.1186/s13099-025-00727-y (PMC12243435; doi:10.1186/s13099-025-00727-y)
Supplement: Supplementary file 7 — Supplementary Material 7 [file 13099_2025_727_MOESM7_ESM.docx]

**Profiling of the tumor-associated microbiome in patients with hepatocellular carcinoma**

Christian Schulz^1^, Ramiro Vilchez-Vargas^1^, Elif Öcal^2^, Nadine Koch^1^, Daniel Puhr-Westerheide^2^, Lu Fornés Burnell^2^, Heidrun Hirner-Eppeneder^2^, Julia Benckert^3^, Maciej Pech^4^, Peter Reimer^5^, Chris Verslype^6^, Christiane Kuhl^7^, Albert Tran^8^, Jens Ricke^2^, Peter Malfertheiner^1,9^, Marianna Alunni-Fabbroni^2*^

^1^Department of Medicine II, LMU University Hospital, LMU Munich, Germany

^2^Department of Radiology, LMU University Hospital, LMU Munich, Germany

^3^Department of Hepatology and Gastroenterology, Charité, Berlin, Germany

^4^Departments of Radiology and Nuclear Medicine, Otto-von-Guericke University of Magdeburg, Magdeburg, Germany

^5^Department of Radiology, Karlsruhe Hospital, Karlsruhe, Germany

^6^Department of Hepatology and Digestive Oncology, University Hospital Gasthuisberg, Leuven, Belgium

^7^Department of Diagnostic and Interventional Radiology, University Hospital, RWTH Aachen University, Germany

^8^Department of Immunology, Université de Nice Sophia-Antipolis, CHU de Nice, France.

^9^Department of Gastroenterology, Hepatology and Infectious Diseases, Otto-von-Guericke University of Magdeburg, Magdeburg, Germany

*Corresponding author

Marianna Alunni-Fabbroni, Department of Radiology, University Hospital, LMU Munich

Marchioninistr. 15, 81377 Munich, Germany, E-mail: marianna.alunni@med.uni-muenchen.de

**Extended Materials and Methods and Results**

*DNA extraction and amplicon library preparation*

Genomic DNA was extracted from formalin-fixed, paraffin-embedded (FFPE) liver tissue samples via the QIAamp DNA FFPE Tissue Kit (Qiagen, Hilden, Germany) according to the manufacturer’s instructions. Briefly, 10 µm of each FFPE sample was cut via a standard microtome, after which the first 3 sections were discarded to avoid cross-contamination. After deparaffinization (3 min, 56°C), proteinase K lysis (1 h, 56°C, 1000 rpm) and incubation at 90°C for 1 h to remove the cross-links, the samples were treated with RNase A (2 min, room temperature) and proteinase K (15 min, 65°C). The DNA was subsequently bound to the columns, washed and eluted in 30 µl of elution buffer (Buffer ATE). The V1-V2 region of the bacterial 16S rRNA gene was amplified via PCR via Platinum^TM^ SuperFi II PCR Master Mix (Thermo Fisher Scientific, Darmstadt, Germany) and the primers 27F (5‘ – AGRGTTHGATYMTGGCTCAG – 3‘) and 338R (5´TGCTGCCTCCCGTAGGAGT – 3‘) for 20 cycles as described previously (14). The DNA concentration was measured via the Quant-iT™ PicoGreen™ dsDNA Assay (Thermo Fisher Scientific). The samples were pooled at equal molar concentrations before being purified (QIAquick PCR Purification, Qiagen). The samples were sequenced on an Illumina MiSeq v3 with 300 bp paired-end sequencing. As negative controls, paraffin-only samples prepared at each recruitment center were used, as were all the reagents used for DNA extraction.

*Immunohistochemical analysis of tissue samples*

From FFPE tumor blocks collected before therapy, 4 µm serial tissue sections were cut, dewaxed and rehydrated according to standard procedures (preheating at 60°C; deparaffinization in Neo-Clear, Merck, Darmstadt, Germany; rehydration in a graded series of ethanol and distilled water) and stained with hematoxylin and eosin for determination of tumor and adjacent areas. For IHC analysis, the primary antibodies anti-LPS (mouse monoclonal, clone WN1 222-5, dilution 1:100, Hycult Biotech, Uden, The Netherlands) and anti-LTA (mouse monoclonal, clone 55, dilution 1:50, Hycult Biotech) were applied overnight at 4°C, followed by incubation with the secondary antibody (goat anti-mouse IgG H&L/HRP, dilution 1:2000, Abcam, Cambridge, UK). A DAB substrate kit (DAB substrate kit, Cell Signaling Technology, Leiden, The Netherlands) was used as a chromogen. The sections were counterstained with Hemalaun (Merck), dehydrated, and mounted with Neo-Mount (Merck). The slides were scanned via an automated slide scanner (Pannoramic SCAN II, 3D HISTECH, Hungary) at 40x magnification. Identification of the cellular subtypes and bacteria was performed by a board-certified pathologist who was blinded to the patient outcome. The presence of defined LPS and LTA signals throughout the tumor tissue was evaluated as positive, whereas the complete absence of signals was evaluated as negative. Tissues with brown cytoplasmic staining in tumoral or immune cells were considered positive.

*Bioinformatic analysis*

The amplicon sequence variants (ASVs) were obtained using dada2 following the standard pipeline (https://benjjneb.github.io/dada2/tutorial.html). ASVs were automatically annotated using silva database (https://www.arb-silva.de/aligner/). Additionally, each ASV was compared against the type strains NCBI database and was annotated to species if the similarity was >99%. Further, ASVs were grouped into two groups, paraffin and human (see SF1). Within paraffin grouped were the ASVs which origin never was described in the upper and lower human gastrointestinal tract. It should be notice that most of the ASVs were previously detected as a contaminant by Nejman D et al. (Science, 2020; doi: 10.1126/science. aay9189). Diversity indices were calculated using the package vegan of R program.

*Diversity indices and bacterial network in ASVs human set*

A total of 134 bacterial species were assigned to human origin (SF3). The richness of each species varied from 2 (SOR33 and SOR75) and 34 (SOR43) and the frequency of each species varied from 1 (*Veillonella ratti*, among others) and 12 (*Streptococcus mitis* and *Streptococcus oralis*). Further, Spearman correlations were calculated using the packages psych and reshape2 packages in R using Benjamini‒Hochberg false discovery correction and considering for downstream analysis only those species with a rho > 0.4 and a q value of <0.05. For visualising the results, we used the program Cytoscape v. 3.10.1. The network was analysed using the tool “network analysis of Cytoscape”*. After analysing the network, we used the degree (Number of Undirected Edges). The degree is detecting which species interact between them in a ranking manner. For example, *Streptococcus oralis* (Frequency 12) has only degree 1, interacting only with *Veillonella rodentium*, while *Veillonella ratti* (Frequency 1) has a degree of 16, interacting with others 16 bacterial species. We considered for downstream analysis only the species with a degree ≤ 5 (a total of 20 species), analysing the scenario of low level of colonization and low probability of including contaminant. Increasing the species with higher degree in the analysis will assume a highest level of colonization in FFPE samples but also increasing level of including contaminants in the dataset. All downstream analysis will perform re-normalizing the dataset and calculating the percentage of abundance of the sub-set of species or genera per sample.

*(https://manual.cytoscape.org/en/latest/Basic_Expression_Analysis_Tutorial.html)

*PCA, heatmaps and ISA analysis*

After setting up the threshold to degree ≤ 5 (a total of 20 species) and re-normalised the dataset, we used the program Past4 for PCA and ISA analysis and heatmaps generation.

(https://www.nhm.uio.no/english/research/resources/past/downloads/past4manual.pdf)
