# Supplementary figures and images for: Profiling of the tumor-associated microbiome in patients with hepatocellular carcinoma
Source: Gut Pathog. 2025 Jul 10;17:53. doi: 10.1186/s13099-025-00727-y (PMC12243435; doi:10.1186/s13099-025-00727-y)

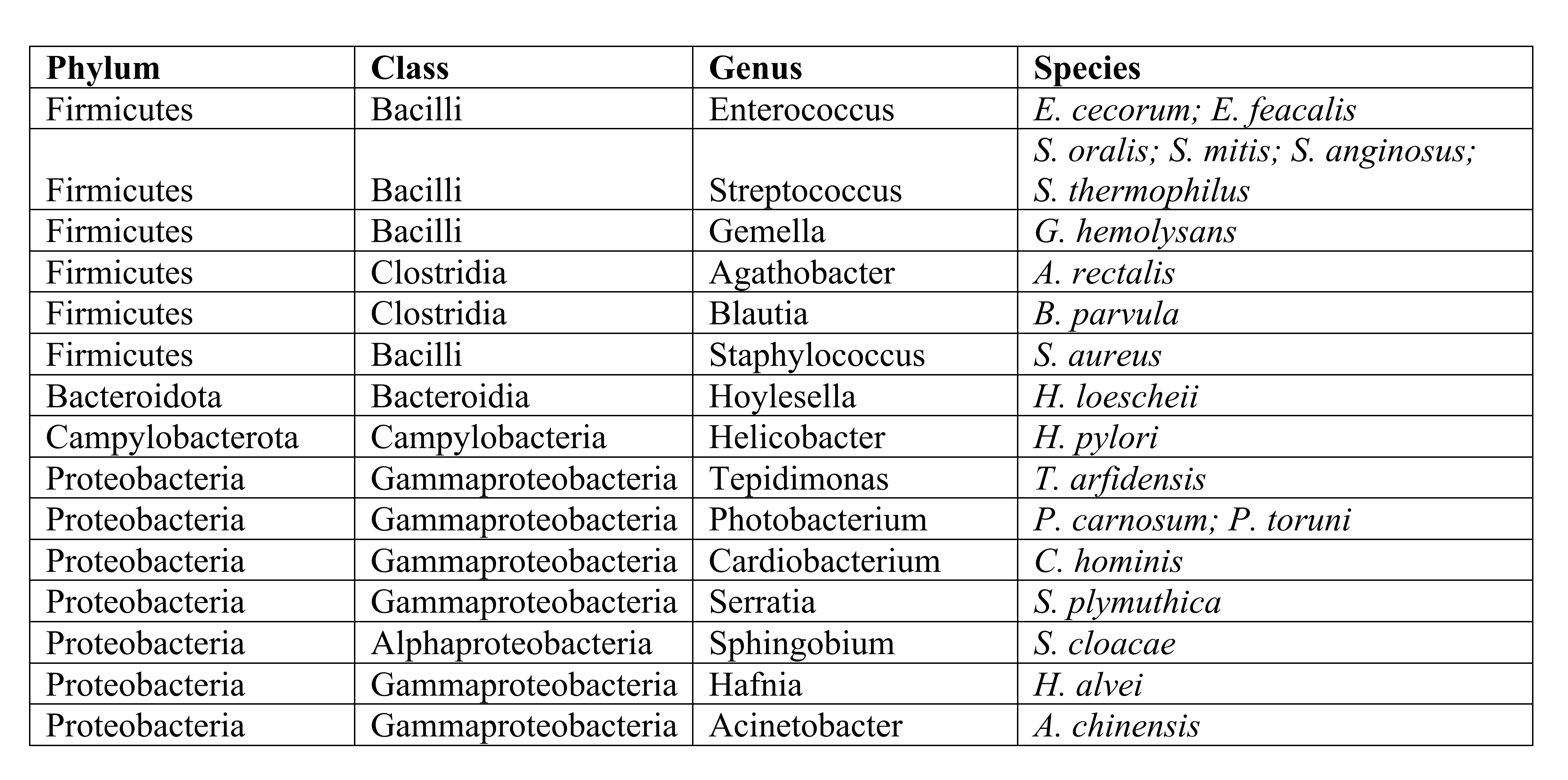

Supplement: Supplementary file 8 — Supplementary Material 8 [file 13099_2025_727_MOESM8_ESM.tiff]
