# Supplementary material for: Profiling of the tumor-associated microbiome in patients with hepatocellular carcinoma
Source: Gut Pathog. 2025 Jul 10;17:53. doi: 10.1186/s13099-025-00727-y (PMC12243435; doi:10.1186/s13099-025-00727-y)

**Supplementary File 8.**  Representative images showing localization of tumor Figure 7 infiltrating lymphocytes in tumor and non-tumor tissues collected from two different clinical cases. (A) Tumoral and non-tumoral tissues collected from patient #62 showed a strong level of inflammation, identified by a high amount of lymphocytes. (B) On the contrary, in both tumoral and non-tumoral tissues collected from patient #15, a lower amount of lymphocytes was visible (yellow arrows). (E, *Enterococcus* - E*. cecorum*; Str, *Streptococcus* - *S. mitis* and *S. oralis*; Sta, Staphylococcus *- S. aureus*)


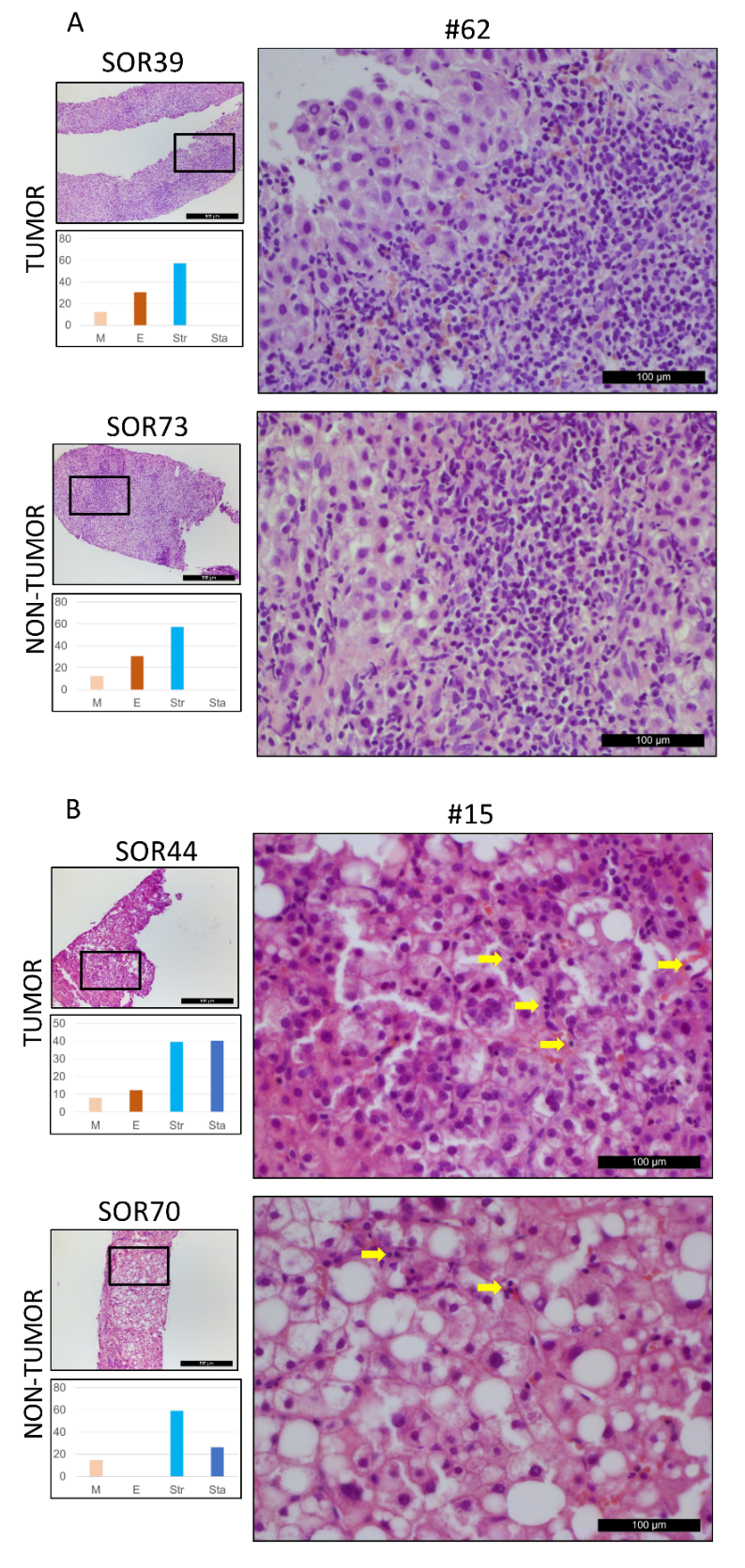

Supplement: Supplementary file 9 — Supplementary Material 9 [file 13099_2025_727_MOESM9_ESM.docx]
